# Molecular mechanisms and functional implications of cyanidin-3-*O*-glucoside interactions with rice starch-protein binary matrix^☆^

**DOI:** 10.1016/j.fochx.2026.103527

**Published:** 2026-01-13

**Authors:** Halah Aalim, Ibrahim Khalifa, Mohammad Rezaul Islam Shishir, Jinyuan Sun, Chenguang Zhou, Xiaobo Zou

**Affiliations:** a Agricultural Product Processing and Storage Lab, School of Food and Biological Engineering, Jiangsu University, Zhenjiang, Jiangsu 212013, China; b China Light Industry Key Laboratory of Food Intelligent Detection & Processing, School of Food and Biological Engineering, Jiangsu University, Zhenjiang 212013, China; c China Food Flavor and Nutrition Health Innovation Center, Beijing Technology and Business University, Beijing 100048, China

**Keywords:** Rice starch, Rice protein, Cyanidin-3-*O*-glucoside, Bioaccessibility, Digestibility, Ternary complex, V-type inclusion complex, Molecular docking

## Abstract

Rice starch-protein interactions significantly influence techno-functional properties and phenolic binding. This study elucidated the molecular mechanisms governing cyanidin-3-*O*-glucoside (C3G) interactions with rice starch–protein matrices containing varying protein levels (0–15% *w*/w). Protein contents of 5% and 10% significantly enhanced C3G binding by 14.8% and 10.7%, respectively, whereas C3G bioaccessibility remained statistically unchanged upon starch digestion. Increasing protein levels reduced particle size, altered granule morphology, and reduced iodine affinity. FTIR analysis revealed strengthened hydrogen bonding, amide-I shifts, and modified starch chain organization, whereas XRD showed reduced crystallinity with attenuated V-type peaks. At 10% protein, C3G produced fine, uniform, pigment-loaded particles, whereas 15% protein induced reaggregation. These structural changes increased resistant starch, decreased slowly digestible starch, and shifted color from red-blue to red-yellow. Molecular docking confirmed that C3G intensified hydrogen bonding and hydrophobic interactions at the starch-protein interfaces. Overall, protein content can be tuned to engineer functional foods with targeted nutritional properties.

## Highlights

• The ternary interactions of rice starch-protein-C3G were examined.
• Protein levels regulated C3G binding and structural reorganization in rice starch.
• C3G boost H-bonds, hydrophobic and non-covalent forces at starch-protein interface.
• A 10% protein produced fine, uniform pigment-loaded stable ternary complex.
• Functional outcomes included enhanced C3G retention and RS, with major color shift.

## 1 Introduction

Anthocyanins, a class of water-soluble flavonoids, impart pigmentation to foods and confer health-promoting functions (Aalim et al., 2021; Herrera-Balandrano et al., 2021). Despite these benefits, their intrinsic instability limits their practical use in functional foods. Anthocyanins are highly susceptible to degradation due to variations in pH, temperature, light exposure, processing conditions, and enzymatic digestion (Aalim et al., 2025; Chen et al., 2019). Consequently, only a small proportion of ingested anthocyanins typically reach the systemic circulation (Aalim et al., 2024). Given these limitations and opportunities, enhancing anthocyanin stability and bioaccessibility is a critical step in fully harnessing their nutritional potential.

Previous studies have demonstrated that food matrix interactions significantly affect the stability and bioaccessibility of anthocyanins. In the rice matrix, starch and proteins are the major constituents, and their hydrogen bonding, electrostatic, and hydrophobic interactions modulate the technical properties and digestibility (Park et al., 2021; Zhuang & Li, 2025). Moreover, minor variations in protein content markedly alter water mobility and hydrogel pore structure, thereby affecting starch swelling and enzymatic accessibility in the starch–protein matrix (Park et al., 2021). However, the role of rice starch-protein matrix interactions in anthocyanin binding and bioaccessibility remains unclear, representing a critical knowledge gap.

The interactions among starch, proteins, and phenolic compounds, including anthocyanins, have been extensively studied as natural approaches for stabilizing bioactive molecules. While the binary interactions of starch–anthocyanins (Li et al., 2024), protein-anthocyanins (Khalifa et al., 2018), and starch–proteins (Zhuang & Li, 2025) have been examined individually, in rice-based foods, starch and protein coexist as the dominant macronutrients. In rice, crude protein accounts for 6–15% of the grain (Amagliani et al., 2017), whereas cyanidin-3-*O*-glucoside (C3G) is the major phenolic compound in black rice (Aalim et al., 2021; Aalim et al., 2024), and their interaction during cooking influences phenolic stability and starch digestibility. Previous studies have shown that rice cooking enhances the intestinal bioavailability of monomeric anthocyanins through starch-anthocyanin associations (Aalim et al., 2024); however, the specific regulatory role of rice proteins in these interactions remains poorly understood. Hence, this study aimed to elucidate the molecular mechanisms underlying the binding of C3G to the rice starch-protein binary matrix at varying protein concentrations (0%, 5%, 10%, and 15%) at a neutral pH of 7.0. This experimental design minimizes compositional variability and enhances mechanistic clarity under physiological and processing conditions. Furthermore, this study investigated the resulting structural and functional modifications of starch-protein systems to provide insights into anthocyanin stabilization and inform the development of rice-based delivery systems for functional food applications.

## 2 Materials and methods

### 2.1 Materials

C3G (Cyanidin 3-*O*-β-glucopyranoside chloride, ≥ 98% purity) was obtained from Shanghai Tauto Biotech Co., Ltd. (Shanghai, China). α-amylase (3000 U/mL) and amyloglucosidase (3300 U/mL) were obtained from Megazyme (Bray, Ireland). Fluorescein isothiocyanate (FITC) and Fast Green were obtained from Macklin (Shanghai, China). Rice starch, characterized by a starch content of 92.57 ± 1.50%, amylose content of 15.71 ± 0.11%, and moisture content of 10.67 ± 0.41%, was obtained from Yuanye Biotechnology Co., Ltd. (Shanghai, China). Defatted rice bran was acquired commercially from Xi ‘an Haoyu Kangze Biological Technology Co., Ltd. (Xi'an, Shaanxi, China).

### 2.2 Protein isolation

Rice protein was isolated using a previously described method (Wang et al., 2018). Defatted rice bran was mixed with distilled water at a solid-to-liquid ratio of 1:10 (*w*/*v*) and magnetically stirred to obtain a homogeneous suspension. The pH of the suspension was adjusted to 12 using 1 M NaOH and continuously stirred for 4 h to facilitate protein extraction. The suspension was centrifuged at 7000 ×*g* (10 min at 4 °C) to separate the supernatant. The supernatant was then subjected to isoelectric point precipitation at pH 4.5 using 1 M HCl solution. The resulting precipitate was collected by centrifugation at 7000 ×*g* (10 min at 4 °C). The precipitate was washed with distilled water, resuspended in approximately five times its weight of distilled water, and neutralized to pH 7 using 0.1 M NaOH. The final slurry was freeze-dried to obtain crude rice protein isolates, which were stored at −20 °C until further use. The crude rice protein isolates were characterized by a protein content of 52.3 ± 0.3% (dry basis), as determined by the Kjeldahl method (N% × 5.95).

### 2.3 Preparation of starch-protein and C3G complexes

The isolated rice protein was mixed with rice starch suspension (1 g, 2.5% *w*/*v*) at varying ratios of 0%, 5%, 10%, and 15% (*w*/w). The mixture was stirred overnight using a magnetic stirrer until solubilization of the protein. The samples were then gelatinized in a boiling water bath for 30 min and allowed to cool to room temperature for further calibration. Subsequently, C3G was added at a concentration of 2 mg/g starch (w/w) to the starch slurry. The pH of all samples was maintained at 7.0 ± 0.1 throughout the process. The mixture was homogenized thrice at 10,000 rpm, incubated at 4 °C overnight, and centrifuged at 5000 ×*g* (20 min at 4 °C). The pellet was washed twice with deionized water, and the supernatants were collected. The samples were coded as follows: RS0, RS5, RS10, and RS15 represented starch-protein-C3G systems with 0%, 5%, 10%, and 15% protein, respectively. To ensure consistency and establish a baseline for comparison, control samples of starch-protein systems, designated as RS0B, RS5B, RS10B, and RS15B, corresponding to samples containing 0%, 5%, 10%, and 15% protein, respectively, were prepared under identical conditions. The precipitates were lyophilized and stored at −20 °C until further analysis.

### 2.4 Determination of C3G binding ratio

The binding ratio of C3G to the rice starch-protein matrix was assessed using a colorimetric method (Li et al., 2024). The total C3G content in the samples was quantified following the precipitation of protein and starch in the supernatants using 1 M HCl. The resulting solutions were centrifuged for 10 min at 10,000 ×*g* and 4 °C. The absorbance of C3G was measured at 520 nm using a UV–Vis microplate spectrophotometer (Thermo Fisher Scientific, Vantaa, Finland). A standard calibration curve for pure C3G was established under identical conditions to correlate the absorbance with the concentration.

### 2.5 In vitro starch digestibility and C3G bioaccessibility

The starch digestibility in starch-protein complexes was assessed using a previously described method (Aalim et al., 2021). Complex samples (100 mg starch, dry weight basis) were suspended in 9 mL of acetate buffer (0.2 M, pH 6.0, containing 200 mM CaCl₂, and 0.5 mM MgCl₂) and equilibrated at 37 °C. Subsequently, 1 mL of a mixed enzyme solution containing 10 U/mL α-amylase and 18 U/mL amyloglucosidase was added to initiate hydrolysis. Aliquots (100 μL) of the enzymatic hydrolysate were collected at predetermined time intervals (0, 20, and 120 min) and immediately mixed with 100 μL of 95% ethanol (*v*/v) to halt the enzyme activity. The unreacted starch residue was separated by centrifugation at 2000 ×*g* for 5 min. The bioaccessibility of C3G was assessed using the colorimetric method described in Section 2.4. The glucose concentration in the supernatant was quantified using the GOPOD assay, and the starch nutritional fractions, rapidly digesting starch (RDS), slowly digesting starch (SDS), and resistant starch (RS), were categorized as follows:

(1) $$\mathrm{RDS}\;\left(\%\right)=\left(\left(G20-G0\;\right)\times 0.9/\mathrm{TS}\;\right)\times 100$$

(2) $$\mathrm{SDS}\;\left(\%\right)=\left(\left(G120-G20\;\right)\times 0.9/\mathrm{TS}\;\right)\times 100$$

(3) $$\mathrm{RS}\;\left(\%\right)=100-\mathrm{RDS}-\mathrm{SDS}$$

where G0, G20, and G120 represent glucose concentrations at 0, 20, and 120 min, respectively, and TS represents total starch.

### 2.6 Characterization of starch-protein and starch-protein-C3G systems

#### 2.6.1 Iodine-binding capacity of starch

The formation of the amylose‑iodine complex was evaluated as previously described Zhang et al., 2023). Briefly, 0.1 mL of the starch–protein complexes was combined with 0.1 mL of iodine reagent (containing 0.08% I₂ and 0.8% KI in water) and allowed to stand at room temperature for 15 min to facilitate complete interaction. The mixture was then diluted to a final volume of 10 mL with distilled water. The absorbance spectrum of the resulting solution was measured in the wavelength range of 300–900 nm using a UV–visible spectrophotometer (UV-1601, Rayleigh, Beijing, China). A blank control was prepared by replacing the samples with an equivalent C3G content to account for any background absorbance.

#### 2.6.2 Colorimetric analysis

The color variations in the starch-protein systems were assessed using a colorimetric spectrophotometer (Model CM2300d, Konica Minolta Holding, Inc., Japan). The results were reported in the CIE Lab* space, where L* denotes lightness (0 = black, 100 = white), a* represents the red–green axis (positive values towards red), and b* the yellow–blue axis (positive values towards yellow). Adobe Photoshop CS6 (version 13.0, Adobe Systems, Inc., San Jose, CA, USA) was employed to convert Lab* color space of the dried samples to RGB. Subsequently, the RGB data were entered into Microsoft Office PowerPoint (version 2021) to populate numerous squares measuring 1.524 × 1.524 cm. The total color difference (ΔE) was calculated as follows:

(4) $$\mathrm{\Delta E}=\sqrt[{2}]{{\left(L{*}_{r}-L{*}_{t}\right)}^{2}+{\left(a{*}_{r}-a{*}_{t}\right)}^{2}+{\left(b{*}_{r}-b{*}_{t}\right)}^{2}}$$

where r and t indicate the raw and treated starch matrices, respectively.

#### 2.6.3 Scanning electron microscopy (SEM) analysis

The surface morphologies of the starch-protein complexes were analyzed using scanning electron microscopy (Regulus 8100, Hitachi High-Technologies, Tokyo, Japan) at an accelerating voltage of 2 kV. The samples were mounted on carbon double-sided tape and sputter-coated with gold prior to imaging. Micrographs were acquired at 1000× and 10,000× magnifications with a 5 μm scale bar.

#### 2.6.4 Confocal laser scanning microscopy (CLSM)

The relative positions of the starch and protein granules in the complexes were explored using CLSM (Leica Microsystems Inc., Heidelberg, Germany). Starch and protein were stained with fluorescein isothiocyanate (FITC) (Macklin, Shanghai, China) and Fast Green (Macklin, Shanghai, China), respectively. Briefly, 20 μL of FITC solution (1 mg/mL) was added to 1 mL of the sample solution (1% *w*/*v*). After staining for 10 min, 20 μL of Fast Green (1 mg/mL) solution was added to each sample. The samples were placed in the dark for 10 min to complete the staining and were observed using CLSM.

#### 2.6.5 Particle size analysis

The particle size distributions (PSD) of the samples were determined by laser diffraction using a Litesizer 500 (Anton Paar, Steiermark, Austria). The samples were dispersed in deionized water at 2.5 mg/mL to conform to the instrument's recommended concentration range.

#### 2.6.6 Fourier transform infrared (FTIR) analysis

FTIR analysis was conducted using FTIR spectrometer (Perkin Elmer Frontier, USA). The scanning range was established at 4000–500 cm^−1^. The spectra were acquired at a resolution of 4 cm^−1^ using 16 scans.

#### 2.6.7 X-ray diffraction (XRD) analysis

XRD analysis was conducted to examine the crystallinity and amorphous characteristics using an X-ray diffractometer (D8 Advance, Bruker, Germany) equipped with a Cu-Kα radiation source. The diffraction angle (2θ) was configured to range from 5° to 40° with a scanning rate of 4°/min and step size of 0.02°.

### 2.7 Molecular docking study

Molecular docking of glutelin with starch and C3G was performed to evaluate the binding interactions, stability, and affinity of the resulting complexes, following a previously described method (Khalifa et al., 2018). Docking simulations were performed using MVD v7.0.0 (Molexus Company, Denmark; license number: Ibrahim_Khalifa). The glutelin crystal structure (AF-A0A5E4F037-F1) was extracted from the AlphaFold database (https://alphafold.com). The chemical structure of C3G was extracted from the PubChem database with a CID of 15,719,498 (https://pubchem.ncbi.nlm.nih.gov), and the structure of starch was created using Cambridge Soft ChemBioOffice Ultra software (Version 14.0) (CambridgeSoft). All structures were energy-minimized using the MM2 force field and subsequently optimized using Hartree–Fock calculations (HF/6-31G (d,p)) in GAUSSIAN 09. Prior to docking, water molecules were removed, and hydrogen atoms were added to the protein structures. The ligand-binding sites of glutelin were identified using the site-finding tool in Discovery Studio (Accelrys Software Inc., San Diego, CA, USA). Discovery Studio v2.5 and MOE 2015 were used to visualize the docked complexes. The BEST method was applied to generate high-quality conformations, followed by energy minimization using the conjugate gradient and Quasi-Newton methods. Docking was conducted using the LibDock algorithm, which treated glutelin as a rigid receptor while allowing full ligand flexibility.

### 2.8 Statistical analysis

The experimental data were analyzed using analysis of variance (ANOVA) and are presented as mean ± standard deviation (SD). One-way analysis of variance (ANOVA) and Tukey's multiple comparison tests were performed using SPSS version 20.0 software (IBM Corp., Armonk, NY, USA). All data were checked for normality and homogeneity of variance using the Shapiro-Wilk and Levene's tests, respectively. Statistical significance was set at *P* < 0.05. All analyses were performed in triplicate.

## 3 Results and discussion

### 3.1 C3G binding ratio, and bioaccessibility

**Fig. 1:**
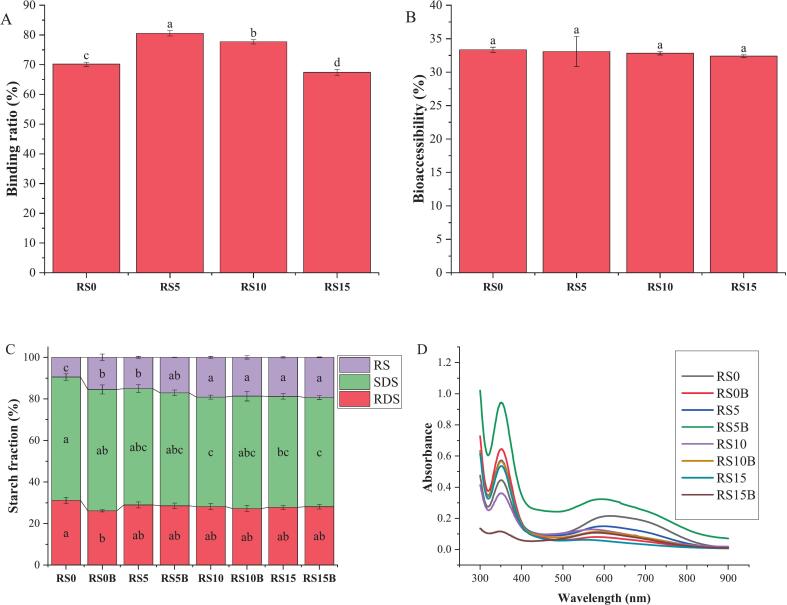
Binding ratio (A), bioaccessibility (B), starch nutritional fractions (C), and starch‑iodine binding spectra (D). Values are expressed as mean ± standard deviation (*n* = 3). Mean values with different superscripts in the same color are statistically different (*P* < 0.05).

The binding of C3G to the starch–protein binary matrices increased significantly with increasing protein content to certain levels, whereas the bioaccessibility of C3G remained unchanged across all formulations during starch digestion. As shown in Fig. 1A, the low protein sample (RS5) demonstrated a significantly superior binding ratio (80.6%) compared to RS0. This binding ratio decreased significantly with increasing protein levels, suggesting that protein levels are crucial for C3G binding. In contrast, Fig. 1B demonstrates that the percentage of C3G released during starch digestion was statistically unchanged across all samples (32.9–33.7, *P* > 0.05), suggesting that protein-C3G bonds were irreversible under starch digestion conditions.

These results highlight the critical role of rice protein in modulating C3G binding in gelatinized starch matrices. The strong protein-C3G interactions are driven by hydrogen bonding, hydrophobic interactions, and electrostatic (Li et al., 2021), provides additional bonding forces beyond the limited binding capacity of starch leading to stabilize the ternary matrix. Moreover, starch gelatinization leads to the development of amylose linear chains, which are crucial for the formation of stable inclusion complexes with anthocyanins (Shen et al., 2025). However, C3G release during starch digestion was unaffected by structural changes in the starch–protein matrix. These findings are consistent with those of a previous study that reported that protein–starch complexes do not influence phenolic bioaccessibility (Ferruzzi et al., 2020). Furthermore, the structural composition and concentration of polyphenols have been shown to significantly influence their interactions with food matrices and digestive enzymes, thereby impacting their release (Aalim et al., 2019; Ferruzzi et al., 2020).

### 3.2 Starch nutritional fractions

The effect of C3G binding on the distribution of starch fractions across various starch-protein binary matrices is depicted in Fig. 1C. The RDS content ranged from 26.1%–31.1%. C3G binding significantly increased RDS in RS0, whereas protein incorporation alone produced a non-significant increase in RDS. These results indicate that C3G may disrupt crystalline domains, potentially enhancing enzymatic hydrolysis. The SDS fraction remained the dominant starch component across all treatments, with values ranging between 52.6 and 59.4%. A slight but consistent increase in SDS content was observed with higher protein levels and C3G inclusion, indicating the formation of partially ordered, slowly digestible domains.

In contrast, RS exhibited a clear increasing trend from RS0 to RS15, implying that the protein and C3G promoted RS formation, likely through retrogradation or crosslinking. However, in RS0, C3G binding significantly reduced RS content, suggesting that anthocyanins disrupt retrograded starch crystallites and destabilize resistant domains. These observations align with previous findings that rice protein reduces starch digestibility by enlarging hydrogel pore sizes and limiting enzyme diffusion, thereby affecting starch–phenolic interactions and structural reorganization (Li, Li, et al., 2021; Mejía Terán & Blanco-Lizarazo, 2021; Zhuang & Li, 2025).

Moreover, the starch–protein complex demonstrated inhibitory effects on both α-glucosidase and α-amylase through a reversible competitive inhibition mechanism, with a stronger effect observed against α-glucosidase (Wang et al., 2019). Additionally, C3G exhibited a modest ability to inhibit α-amylase, an effect attributed to its bulky glycosylated moiety, which hindered active-site binding, limited hydroxylation of its B-ring, and the absence of stabilizing methyl groups (Xiao et al., 2025). Collectively, these findings indicate that the combined presence of protein, starch, and C3G exerts synergistic effects, simultaneously promoting RS formation and modulating C3G release.

### 3.3 Starch‑iodine binding

The absorbance spectra of the different samples (Fig. 1D) displayed distinct variations in intensity and peak shifts, providing insights into the structural changes in starch systems. RS0, RS5, and RS5B showed the highest λ-max (red shifts) with a broad peak and high intensity centered at approximately 600 nm, indicating a strong amylose‑iodine helical inclusion complex and confirming the presence of amylose helices. Moreover, the presence of a pronounced broad peak suggests increased heterogeneity in the amylose chain and effective iodine binding facilitated by C3G and protein-starch interactions (Amoako & Awika, 2019).

In contrast, the absorbance intensities of RS0B, RS10, RS10B, RS15, and RS15B showed significant blue shifts, and the reductions intensified with C3G binding. This indicates a decline in iodine-binding affinity, likely due to the significant loss of helical integrity and structural disruptions (Zhang et al., 2024) caused by the structural arrangement of protein and C3G binding. The lower absorbance of RS10 and RS15 suggests that C3G elicited higher modification levels, leading to more pronounced changes in amylose helices, further diminishing iodine complexation.

### 3.4 Physical appearance and colorimetric properties

**Fig. 2:**
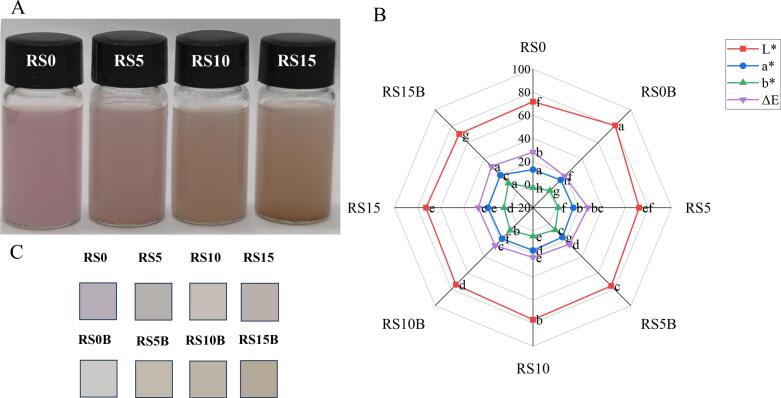
Strach-protein-C3G complex appearance (A), colorimetric values (B), and RGB chrominance values (C) of the samples. Values are expressed as mean ± standard deviation (n = 3). Mean values with different superscripts in the same color are statistically different (*P* < 0.05).

The incorporation of C3G and protein into the starch matrix resulted in significant changes in colorimetric values. Fig. 2(A–C) illustrates the appearance of the C3G complex, accompanied by the RGB and CIE Lab* values of the dried samples. As shown in Fig. 2A, increasing protein content displayed progressive color changes from pink to tan/brown and relatively consistent turbidity across samples. With C3G binding, the L* values remained relatively stable (71.8–72.7), with a slight peak in lightness at 10% protein (77.0). The redness index (a*) showed a clear downward trend from +4.26 at RS0 to +1.38 at RS15, indicating a progressively reduced red coloration with increasing protein content. Conversely, the yellowness index (b*) shifted from a mild blue tint (b* = −2.78) at RS0 to a yellow color (b* = +5.14) at RS15. The total color difference (ΔE) decreased from 28.14 (RS0) to a minimum of 23.09 at RS10 and increased again to 27.33 at RS15.

In the blank samples, RS0B exhibited the highest lightness (L* = 80.72) and lowest ΔE (18.81), with almost no red shift (a* = −0.12) and only a slight yellow tint (b* = +0.94). With an increasing protein ratio to 15%, L* declined steadily (70.33), whereas b* rose sharply (+10.05) in RS15B, reflecting a strong yellowing effect. The redness index first rose modestly to +1.0 at RS5B and then increased again to +1.87 at RS15B but remained far higher than the RS0B value. ΔE increased continuously from 18.81 (RS0B) to 30.78 (RS15B), indicating that high protein binding incurs a greater overall perceptual color change compared to native starch.

The observed chromatic alterations in the treated matrices correspond with the documented effects of rice grain gelatinization and C3G complexation, as reported in the literature (Aalim et al., 2021; Hu et al., 2024; Zhou, Li, et al., 2024). In starch-based matrices, the restricted mobility of water molecules and protons in proximity to the chromophore reduces the intensity of red–blue vibrancy (Yang et al., 2021), thereby influencing the observed pigmentation patterns.

### 3.5 Morphological analysis

**Fig. 3:**
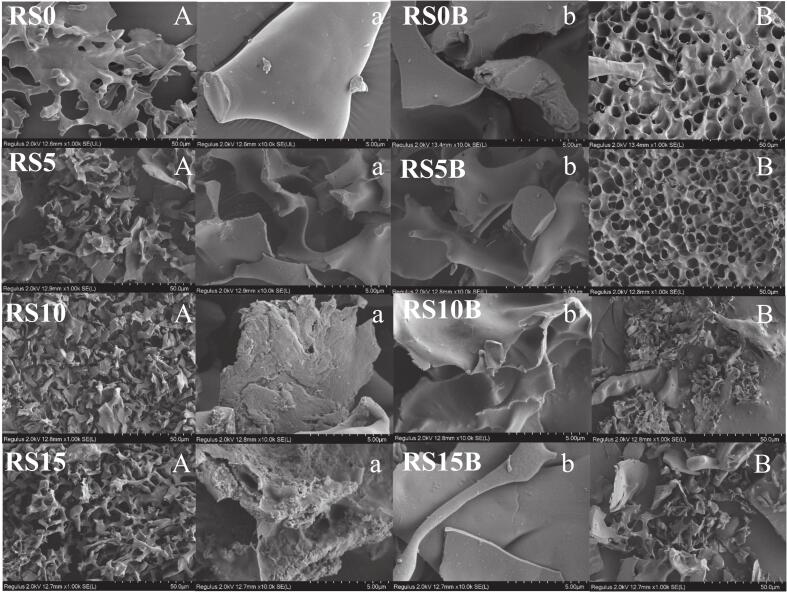
The microstructural characteristics of the samples were examined using scanning electron microscopy at 1000× (A and B) and 10,000× (a and b) magnifications.

Morphological examination by SEM (Fig. 3) revealed a clear concentration-dependent reconfiguration of the gelatinized starch–protein microstructure upon C3G binding. Gelatinized starch (RS0B) exhibited a porous, sheet-like network with relatively smooth surfaces, a morphology consistent with complete starch gelatinization (Dai et al., 2023). In contrast, the C3G-treated counterpart (RS0) was noticeably denser, with thick lamellae and a rough surface. This suggests a surface association between starch and C3G, along with a partial collapse of the fine porosity. The addition of protein amplified these effects. In the ternary starch–protein–C3G systems, RS5 and RS10 developed increasingly fragmented, crumpled granule remnants, and rougher surfaces, while RS15 displayed a densely aggregated microstructure consistent with protein overloading and extensive structural disruption. In starch–protein systems, the morphology shifted from a uniform, sponge-like porous network with embedded protein inclusions at low protein content (RS5B) to irregular, compact, and heterogeneous aggregates at moderate and high protein concentrations (RS10B, RS15B).

Collectively, the SEM images suggest that C3G and protein play a dual role in altering starch structure. Lower protein levels promote network stabilization and pore formation, likely via hydrogen bonding and hydrophobic or aromatic interactions (Mejía Terán & Blanco-Lizarazo, 2021). Elevated protein levels facilitate aggregation and crosslinking driven by intensified protein-protein interactions through hydrophobic interactions, hydrogen bonding, electrostatic shielding, and disulfide bonds (Amagliani et al., 2017; Hou et al., 2017; Wang et al., 2018). Furthermore, elevated protein levels induce lamellar collapse and surface heterogeneity, characterized by steric masking and bridging (Zhuang & Li, 2025). These structural changes plausibly underlie the observed alterations in functional behavior, including changes in digestibility, particle size, and pigment retention.

### 3.6 Confocal laser scanning microscopy analysis

**Fig. 4:**
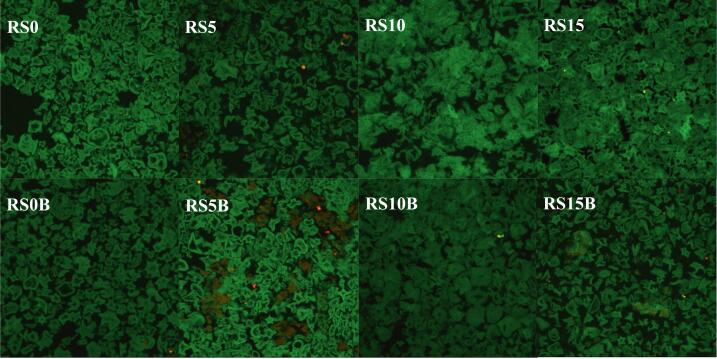
Confocal laser scanning micrographs of the samples, with gelatinized starch molecules (green), and proteins (red). (For interpretation of the references to color in this figure legend, the reader is referred to the web version of this article.)

Fig. 4 presents confocal micrographs of the tested samples, illustrating gelatinized starch molecules stained with a green dye and proteins stained with a red dye. CLSM of rice starch-protein-C3G matrices revealed that increasing the protein content correlated with more clustered and aggregated fluorescence patterns, indicating progressive structural changes. The corresponding blank samples consistently amplified these effects at each protein level, resulting in increased sample heterogeneity. The marked textural contrast suggests significant structural rearrangements at the increasing protein levels.

Interestingly, the gelatinized starch samples (RS0 and RS0B) showed significant protein fluorescence, which was attributed to the innate proteins of starch granules (Han & Hamaker, 2002). Furthermore, the results revealed a decrease in protein fluorescence with increasing protein concentration upon C3G binding. This was primarily attributed to anthocyanin-induced static fluorescence quenching, a well-documented phenomenon in which C3G binds to protein molecules and forms non-fluorescent ground-state complexes (Chen et al., 2023). Spectroscopic analyses of various proteins, such as bovine serum albumin, β-lactoglobulin, and walnut protein isolate, have consistently demonstrated that C3G engages in hydrophobic interactions and hydrogen bonding. This interaction alters the secondary structure of the protein, characterized by a reduction in α-helix content and an increase in β-sheet and random coil formation (Tang et al., 2016). These observations suggest an increase in the hydrophilicity of the protein microenvironment upon binding (Ren & Giusti, 2021).

### 3.7 Particle size distribution

**Fig. 5:**
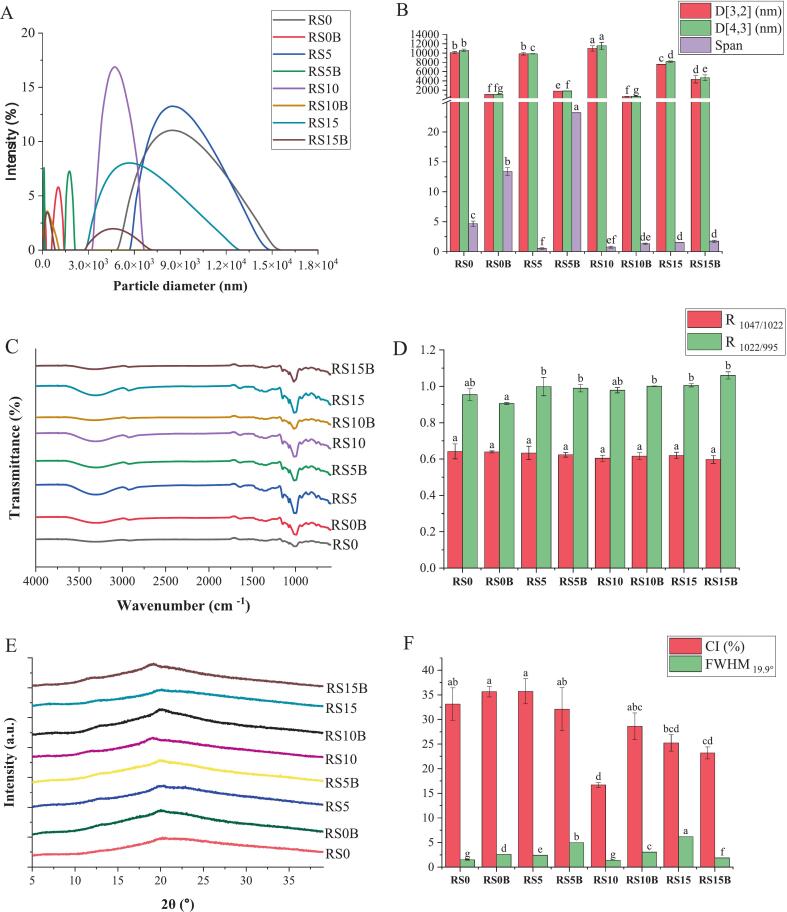
Particle size distributions (A), surface-weighted D [3,2], volume-weighted D [4,3] mean diameters, and volume span (B), FTIR spectra (C), FTIR band-intensity ratios (D), X-ray Diffraction pattern (E), and crystallinity index (CI) and full width at half maximum (FWHM) of the characteristic peak at 19.9° 2θ (F). The results are presented as mean ± SD (n = 3). Mean values with different superscripts in the same color are statistically different (*P* < 0.05).

Intensity-based particle size distribution (PSD) curves and mean diameters, specifically the surface-weighted D[3,2] and volume-weighted D[4,3], along with the span of the samples, are illustrated in Fig. 5A and B. Increasing protein levels resulted in a progressive reduction in particle size upon C3G binding, suggesting increased granule disintegration. The PSD curves for RS0 and RS5 displayed dominant peaks between 5.23 and 14.98 μm. RS0 had mean diameters of D[3,2] = 10.08 μm and D[4,3] = 10.59 μm with a span of 4.66, indicating a moderately broad distribution of particle sizes. In contrast, RS5 showed slightly smaller mean sizes D[3,2] = 9.85 μm and D[4,3] = 9.84 μm with a much narrower span (0.51), which is consistent with a more uniform population. These findings suggest a homogeneous population, with broader distributions potentially attributable to the aggregation or formation of amylose-C3G complexes (Du et al., 2022).

RS10 displayed a pronounced shift towards smaller, more heterogeneous granules, with a modal peak between 3.49 and 6.14 μm, yet D[3,2] and D[4,3] were 11.00 μm and 11.56 μm, respectively. The disparity between the modal peak and elevated mean diameters indicates a skewed distribution, in which a dominant population of small granules coexists with a minority of larger aggregates that disproportionately inflate the weighted averages. In contrast, RS15 showed a clear bimodal distribution with peaks at 0.32–0.61 μm and 2.96–12.74 μm, and mean sizes of D[3,2] = 7.58 μm and D[4,3] = 8.13 μm (span = 1.51), indicating simultaneous fragmentation into fine particles and aggregation of larger fragments at higher protein levels. Notably, C3G has been reported to increase starch particle size at neutral pH (Li, Li, et al., 2021), which could further account for the elevated D[3,2] and D[4,3] values observed despite the presence of small modal populations.

Gelatinization of the starch–protein matrices resulted in pronounced granule fragmentation and multimodal PSDs, with a decrease in the overall mean diameter and an increase in heterogeneity (span) as the protein content increased. The effect was non-linear, and low protein content (RS0B and RS5B) resulted in broad, heterogeneous aggregates with incomplete starch surface coverage. Intermediate protein (RS10B) produced the smallest and most uniform particles (D[3,2] = 0.595 μm; D[4,3] = 0.687 μm; span = 1.29), which is consistent with optimal surface stabilization of the fragments. However, at the highest protein level (RS15B), the mean sizes rebounded (D[3,2] = 4.32 μm; D[4,3] = 4.66 μm; Span = 1.73), indicating the saturation of binding sites and the onset of protein-mediated reaggregation. Such aggregation may hinder enzyme accessibility and, consequently, diminish starch digestibility (Liang et al., 2021).

### 3.8 FTIR and short-range ordered structure

FTIR spectra (Fig. 5C) revealed distinct chemical and structural changes in starch-protein matrices following C3G binding, with similar absorption bands and functional groups to gelatinized starch. The broad absorption band observed in the 3440–3070 cm^−1^ region, was attributable to intermolecular hydrogen bonding and O—H stretching vibrations (Aalim & Luo, 2021), which is intensified and broadened by C3G binding, particularly in starch-protein matrices. This spectral behavior is consistent with the formation of additional hydrogen bonds between anthocyanin hydroxyls and starch hydroxyls or protein amide groups, and with increased water retention or reorganized hydration shells around the biopolymer network (Su et al., 2025).

In rice crude protein (Fig. S1), the amide-I band was observed at 1650 cm^−1^ arising primarily from C=O stretching of the peptide backbone, while the amide-II region shoulder peak was observed near 1540 cm^−1^ associated with N—H bending and C—N stretching (Dai et al., 2023). In starch-protein matrices, the amide-I band shifted to approximately 1630–1640 cm^−1^ and subsequently intensified following C3G binding, while the amide-II band was diminished. The slight downshift and broadening of the amide I band suggest a disruption in the protein secondary structure, characterized by a reduction in the ordered α-helical content and a relative increase in the β-sheet and random coil populations (Hou et al., 2017).

The carbohydrate fingerprint region 900–1500 cm^−1^ (Aalim et al., 2024) showed significant variations. The characteristic starch C—O—C and C—O stretching bands at 1047, 1022, and 995 cm^−1^ were present across samples but displayed altered intensities and slight bandwidth shifts upon C3G binding and protein interaction. This observation was consistent with the conformational rearrangements induced by C3G (Zhai et al., 2017). Two emergent bands near 1445 cm^−1^ in the starch-protein-C3G matrices were assignable to the aromatic C=C stretching of the cyanidin aglycone, consistent with anthocyanin incorporation into the matrix helices via hydrogen bonding (Zhang, Hassane Hamadou, Chen and Xu, 2023).

**Fig. 6:**
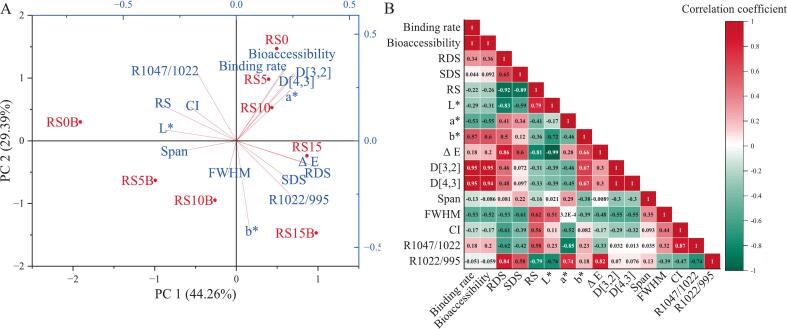
PC scores and loading plot (A) and Pearson correlation coefficients of the variables and observations (B).

Finally, quantitative ratio analysis (Fig. 6D) of the starch order marker bands R_1047/1022_ showed non-significant variations among the samples, suggesting that C3G binding and protein complexation on the gelatinized starch did not disrupt the short-range molecular order (Zhou et al., 2024). The helix index (R_1022/995_) showed a marginal increase after C3G and protein complexation, reflecting a minor increase in residual double helices (Zhou, Zhou, et al., 2024), are consistent with the observed increase in RS formation.

### 3.9 XRD and crystalline structure

The XRD patterns, crystallinity index (CI), and full width at half maximum (FWHM) of the characteristic peak at 19.9° 2θ for various samples are presented in Fig. 6E and F. Starch gelatinization eradicated the native A-type diffraction pattern and formed a modest V-type structural peak centered at 19.9° 2θ. This peak was consistent with the formation of endogenous amylose-helix inclusion motifs in both the binary starch–protein matrices and the C3G-bound samples. A significant reduction in CI was observed with increasing protein content, decreasing from 35.64% in the RS0B matrix to 16.69% in the RS10 matrix. This loss of long-range order is attributed to protein chains intercalating into starch double helices during gelatinization, disrupting hydrogen-bonded lamellae, and generating amorphous, protein-rich domains (Zhuang & Li, 2025).

The binding of C3G further altered the V-type diffraction pattern. The V-type peak exhibited a reduced FWHM after C3G binding, consistent with the penetration of C3G into the starch–protein interfacial regions and interference with starch helix stacking. At higher protein contents (RS10 and RS15B), the V-peak shifted slightly to 19.0° 2θ, an angle change compatible with minor lattice expansion caused by both C3G and protein insertion between starch chains and the formation of hydrogen bonds with starch hydroxyls and protein amide groups (Zhang et al., 2023; Zhuang & Li, 2025). Such insertion weakens the cohesive forces that stabilize the crystalline lamellae and accounts for the observed reduction in the CI.

These observations align with previous reports indicating that proteins or protein hydrolysates can reduce starch crystallinity by inhibiting retrogradation and increasing the amorphous fraction (Dai et al., 2023). Anthocyanins may promote V-type ordering via amylose–polyphenol inclusion complexes, thereby modulating digestibility (Zhang et al., 2023). Proteins can also bind to anthocyanins through hydrophobic and hydrogen bonding interactions, potentially competing with starch-binding sites and altering pigmentation and polysaccharide crystallinity (Wu et al., 2023).

### 3.10 Principal component analysis

Principal component analysis (PCA) was conducted on the physicochemical and functional parameters to reduce dimensionality and reveal multivariate patterns (Fig. 6A). PCA was applied to 16 quantitative descriptors measured in the samples, including the binding ratio, colorimetric parameters (L*, a*, b*, ΔE), particle size measures (D [3,2], D [4,3], Span), crystallinity indicators (CI, R_1047/1022_, R_1022/995_, FWHM), and digestibility and bioaccessibility indices (RDS, SDS, RS, bioaccessibility). The first two principal components (PC1 and PC2) accounted for 73.65% of the total variance, indicating that PC1 (44.26%) and PC2 (29.39%) captured most of the systematic variation across the dataset. Loadings showed that PC1 was dominated by shifts in starch nutritional fractions, L*, ΔE, Span, and CI, whereas PC2 primarily reflected C3G binding, bioaccessibility, redness, yellowness, FWHM, particle mean diameters (D[3,2], D[4,3]), and infrared ratios (R_1047/1022_, R_1022/995_).

In the score plot, samples with similar C3G-binding and bioaccessibility behaviors (RS0, RS5, and RS10) clustered in positive PC1 and PC2, highlighting the role of C3G binding in driving sample characteristics, which were characterized by larger mean diameters and increased redness. Increasing protein levels within this group produced a modest downward drift in PC2, consistent with incremental changes in molecular disorder. In contrast, the high-protein samples (RS15 and RS15B) were distributed in the positive-PC1 and negative-PC2 quadrants. RS15 was significantly correlated with increased ΔE, RDS, and SDS and a higher R_1022/995_ ratio, whereas RS15B was associated with enhanced yellowness (b*). These findings suggest that elevated protein levels influence the characteristics indicative of altered pigmentation, digestibility, and modified short-range order. Notably, the mid-protein blank matrices RS5B and RS10B were dispersed on negative PC1 and PC2, signifying a close influence on the Span and FWHM, respectively. Finally, RS0B was loaded high on negative PC1 and low on positive PC2, showing a low influence on the infrared ratio R_1047/1022_, CI, RS, and color lightening (L*).

Mechanistically, PCA clustering and loading support the coherent structure-function interplay. The protein content modulated C3G binding to gelatinized starch, and this ternary interaction simultaneously interchanged starch digestibility, changed matrix crystallinity, and caused color changes (PC1), while producing a non-linear effect on molecular disorder, particle size, and increasing color yellowness (PC2), which was strongest at higher protein levels.

### 3.11 Pearson correlation analysis

Pearson's correlation analysis of the 16 variables describing the starch-protein and C3G complexes (Fig. 6B) revealed several strong and biologically meaningful relationships between the variables. The C3G binding rate and bioaccessibility were essentially identical (*r* = +1.00, *P* < 0.0001) and both were strongly correlated with the mean particle size (D[3,2] and D[4,3]; *r* ≥ +0.95, *P* < 0.0005). The starch digestive fractions exhibited the expected compromises, and RDS and SDS were strongly and inversely correlated with RS (*r* ≤ −0.89, *P* < 0.003), indicating a shift in starch digestibility linked to C3G and protein complexation.

Color metrics and molecular-order markers were closely associated with the nutritional fractions of starch. For example, RDS correlated negatively with lightness L* (*r* ≤ −0.83, *P* < 0.01) and positively with ΔE and the infrared order ratio R_1022/995_ (*r* ≤ +0.86, *P* < 0.007), whereas RS showed opposite trends with L* (*r* ≤ 0.79, *P* < 0.018) and R_1022/995_ (*r* ≤ −0.79, *P* < 0.019). Lightness L* was negatively associated with redness a* (*r* ≤ −0.72, *P* < 0.05) and nearly perfectly inversely related to the total color change ΔE (*r* ≤ −0.99, *P* < 0.0001). The two particle size measures were effectively identical (D[3,2] vs. D[4,3], *r* = +1.00, *P* < 0.0001). Finally, FTIR-derived markers of crystallinity and helicity showed a moderate negative relationship (*r* = −0.74, *P* < 0.036), whereas R_1047/1022_ correlated negatively with redness b* (*r* = −0.85, *P* < 0.008) and positively with CI (*r* = +0.87, *P* < 0.005). The consistent associations between the FTIR order ratios, CI, and color metrics (L*, b*, ΔE) indicate that the disruption of the short-range order manifests as measurable color shifts.

Collectively, these correlations corroborate the PCA results and map a coherent structure-function landscape of protein interactions and C3G binding. Protein-mediated C3G binding produces larger, darker, and more anthocyanin-rich granule aggregates that are evenly bioaccessible and less ordered at the molecular scale.

### 3.12 Molecular docking

**Fig. 7:**
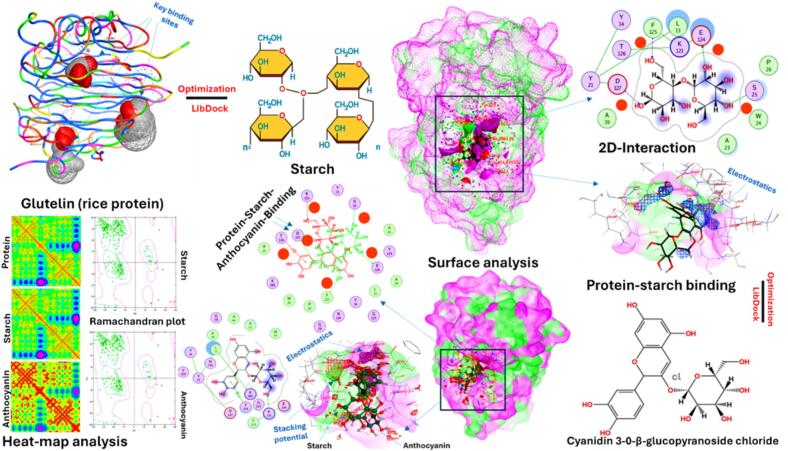
Molecular docking of the interaction between starch, glutelin, and C3G, surface binding analysis, 2D-binding, protein-ligand interaction, heat-map analysis, and Ramachandran plot analysis.

Molecular docking analysis was performed to corroborate the experimental findings regarding C3G binding to starch-protein binary matrices, the results are shown in Fig. 7 and Table 1. Glutelin constitutes over 80% of the protein in rice endosperm (Amagliani et al., 2017) and serves as a highly representative model for the interactions observed with rice protein isolate. The docking scores and binding energies revealed enhanced interactions upon C3G binding. A negative binding energy indicates that the binding process was spontaneous (Yang et al., 2025). Specifically, the glutelin-starch-C3G complex exhibited a superior docking score of 168.3 and a more favorable binding energy of −8.7 kcal/mol than the glutelin-starch complex. A recent study reported that rice protein-C3G conjugates exhibit spontaneous binding at pH 7 with a free energy of −8.2 kcal/mol (Yang et al., 2025), suggesting that the protein enhances the binding energy of the ternary matrix. In contrast, the glutelin–starch complex demonstrated a greater binding affinity than the ternary matrix. This observation aligns with the SEM analyses, which revealed more compact and heterogeneous aggregates with smoother surface morphologies in the starch-protein binary matrices.

**Table 1:** Binding constants for molecular docking analysis of rice starch with glutelin and C3G.

Parameters	Starch-Protein	Starch-Protein-C3G
Docking score	142.8	168.3
Binding energy (kcal/mol)	−7.2	−8.7
Binding affinity	5.1	0.42
Electrostatic energy (μM)	−15.4	−18.9
Van der Waals Energy (kcal/mol)	−8.9	−11.6
Hydrogen bond energy (kcal/mol)	−3.2	−5.8
Hydrophobic interactions (kcal/mol)	−12.1	−15.7
Surface area buried (kcal/mol)	782.5	1124.8
Number of H-bonds (Ų)	6	9
Ramachandran favored (%)	94.2	95.8
Ramachandran allowed (%)	5.1	3.9
Ramachandran outliers (%)	0.7	0.3

Notably, the starch-protein matrix interactions were primarily driven by electrostatic energy and hydrophobic interactions, which were further enhanced upon C3G binding. The increase in the number of H-bonds from 6 to 9 in the ternary matrix elevated the H-bond energy from −3.2 to −5.8 kcal/mol, while simultaneously increasing the van der Waals interactions from −8.9 to −11.6 kcal/mol, further supporting the ternary complex stability. These forces were optimally balanced at RS10, resulting in fine rough particles, high C3G binding, and stable ternary complexes. Additionally, C3G binding enhanced the expansion of the buried surface area by over 342 Å^2^ in the ternary complex, reflecting a denser protein-ligands interface. These results align with our FTIR data, where C3G binding intensified O–H/N–H absorption and perturbed amide-I bands, indicating strengthened hydrogen bonding and altered protein secondary structure.

Surface mapping identified key glutelin amino acids involved in C3G binding, including E124, F125, T126, D127, K121, and S25, which participate in hydrogen bonding and π–π stacking, and hydrophobic pockets around K166, Y14, L13, and A23, which stabilize the starch moiety via van der Waals contacts. Heat-map visualization of residue-level affinity confirmed these hotspots, with warmer clusters along the protein-C3G diagonal demonstrating enhanced contact density compared to protein-starch interactions, highlighting the key regions of cooperative binding. The Ramachandran plot generated for the optimized docking model corroborated these findings by showing that 95.8% of the residues occupied the favored regions, 3.9% fell within the allowed regions, and only 0.3% were outliers, ensuring minimal backbone distortion. This was consistent with the general validation criteria, where high-quality models typically feature >90% of residues in the favored regions. Overall, the Ramachandran plot confirmed that the structural model of the glutelin-starch-C3G complex was stereochemically sound, supporting further interpretation of the molecular binding, interaction energies, and docking-derived functional mechanisms of the complex.

Collectively, these results demonstrate that C3G inclusion reinforced glutelin-starch binding by amplifying noncovalent forces, optimizing surface complementarity, and preserving protein backbone geometry. This synergistic stabilization mechanism suggests that anthocyanins can act as molecular bridges in starch-protein matrices, potentially enhancing the stability, digestibility, and functional performance of rice-based protein-polysaccharide-polyphenol complexes in food applications.

## 4 Conclusion

This study demonstrates that protein levels are pivotal regulators of starch-C3G interactions and the consequent structural and functional properties of rice ternary matrices. Moderate protein levels enhanced C3G binding and reduced granule size and morphology while maintaining comparable C3G bioaccessibility throughout starch digestion. Molecular docking further confirmed that C3G stabilized starch-protein interfacial interactions through hydrogen bonds, hydrophobic contacts, electrostatic energy, Van der Waals Energy, and π–π stacking with key glutelin amino acid residues. An intermediate protein concentration (10%) produced the most uniform and structurally stable, C3G loaded complexes, whereas excessive protein (15%) induced reaggregation and reduced structural coherence. These molecular transitions translated into measurable changes in starch digestibility, promoting resistant starch formation and modulating color attributes. Collectively, these findings provide mechanistic insights into the regulation of anthocyanin binding and bioaccessibility by rice proteins in starch matrices, offering a rational basis for tailoring starch-protein-polyphenol complexes to improve anthocyanin stability, modulate starch digestibility, and design next-generation functional food systems.

## CRediT authorship contribution statement

**Halah Aalim:** Investigation, Funding acquisition, Formal analysis, Data curation, Conceptualization. **Ibrahim Khalifa:** Writing – review & editing, Software, Data curation. **Mohammad Rezaul Islam Shishir:** Writing – review & editing, Investigation. **Jinyuan Sun:** Resources, Methodology. **Chenguang Zhou:** Writing – review & editing, Validation, Supervision, Resources, Project administration, Funding acquisition. **Xiaobo Zou:** Writing – review & editing, Supervision, Resources, Project administration, Funding acquisition.

## Declaration of competing interest

The authors declare that they have no known competing financial interests or personal relationships that could have appeared to influence the work reported in this paper.

## Data Availability

Data will be made available on request.

## Appendix A Supplementary data

Supplementary material Image 1
