# Supplementary material for: Molecular mechanisms and functional implications of cyanidin-3-O-glucoside interactions with rice starch-protein binary matrix
Source: Food Chem X. 2026 Jan 13;33:103527. doi: 10.1016/j.fochx.2026.103527 (PMC12853059; doi:10.1016/j.fochx.2026.103527)
Supplement: Supplementary file 1 — Supplementary material [file mmc1.docx]

Supplementary materials

Molecular mechanisms and functional implications of cyanidin-3-*O*-glucoside interactions with rice starch-protein binary matrix

Halah Aalim^ab^, Ibrahim Khalifa^ab^, Mohammad Rezaul Islam Shishir^ab^, Jinyuan Sun^c^, Chenguang Zhou^ab^*, Xiaobo Zou^ab^*

^a^ Agricultural Product Processing and Storage Lab, School of Food and Biological Engineering, Jiangsu University, Zhenjiang, Jiangsu 212013, China

^b^ China Light Industry Key Laboratory of Food Intelligent Detection & Processing, School of Food and Biological Engineering, Jiangsu University, Zhenjiang 212013, China

^c^ China Food Flavor and Nutrition Health Innovation Center, Beijing Technology and Business University, Beijing 100048, China

*Corresponding author.

Xiaobo Zou, PhD, Professor & Chenguang Zhou, PhD, Assoc. Professor

Agricultural Product Processing and Storage Lab, School of Food and Biological Engineering, Jiangsu University, Zhenjiang, Jiangsu, 212013, China

Email: [zou_xiaobo@ujs.edu.cn](mailto:zou_xiaobo@ujs.edu.cn); [zhouchenguang@ujs.edu.cn](mailto:zhouchenguang@ujs.edu.cn)


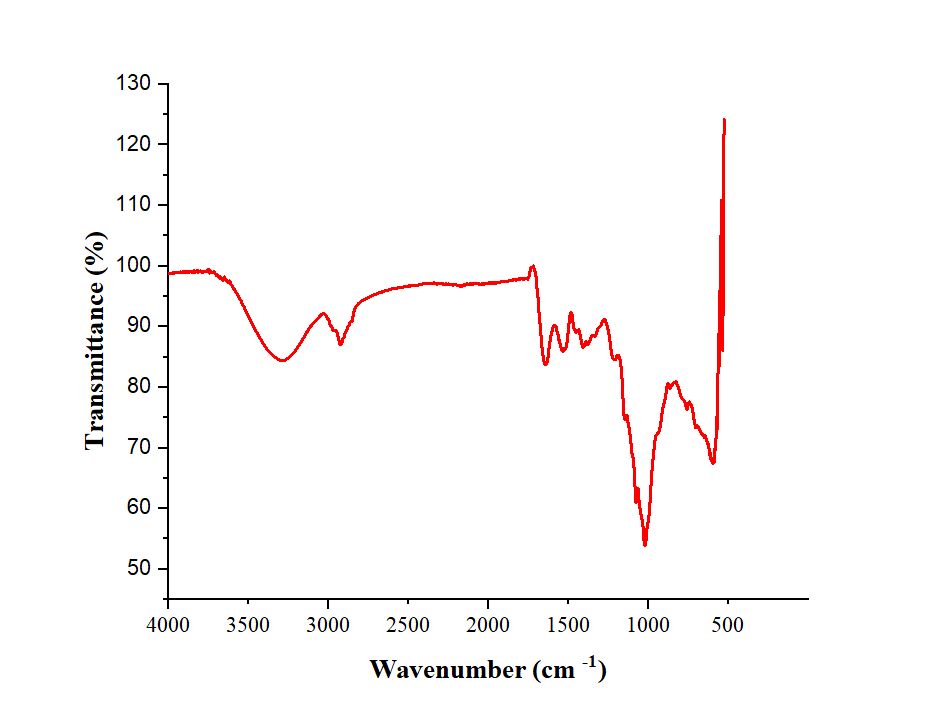


Figure S1. FTIR spectra of isolated rice bran protein.
